# Applying knowledge and experience from potato (*Solanum tuberosum*) to update genetic stability data requirements in the risk assessment for vegetatively propagated biotech crops

**DOI:** 10.3389/fbioe.2024.1376634

**Published:** 2024-04-04

**Authors:** Matthew G. Pence, Muffy Koch, Jaylee DeMond, Gary Rudgers

**Affiliations:** Simplot Plant Sciences, J. R. Simplot Company, Boise, ID, United States

**Keywords:** genetic stability, vegetative propagation, regulation, risk assessment, potato

## Abstract

Regulatory agencies require data on genetic stability as part of the safety assessment for biotech crops, even though the genetic stability of a plant is not necessarily an environmental, human or animal health safety concern. While sexual reproduction has the potential to introduce genomic variation in conventionally bred and biotech crops, vegetative propagation is genetically stable. In vegetatively propagated crops, meiosis does not occur thus limiting the number of homologous recombination events that could lead to chromosomal rearrangements in progeny plants. Genetic stability data is often, but should not be, an automatic requirement for the safety assessment of vegetatively propagated biotech crops. Genetic stability data from biotech potato events has demonstrated that vegetative propagation of potato tubers does not affect the stability of introduced DNA sequences or lead to loss of trait efficacy. The knowledge and experience gained from over 30 years of assessing the safety of biotech crops can be used by regulatory authorities to eliminate data requirements that do not address environmental, food or feed safety concerns. As a first step, regulators should consider removing requirements for genetic stability as part of the safety review for vegetatively propagated biotech crops.

## 1 Introduction

Plants naturally evolve, and genetic heterozygosity in plants is due to mutations, transposable elements, homologous recombination, gene silencing, and even whole genome duplication resulting in polyploidy (Flint-Garcia, 2013; Soltis and Soltis, 2021). The ability of plants to evolve has enabled the improvement of domesticated crops using conventional breeding techniques (i.e., crossing between sexually compatible species) (Flint-Garcia, 2013). Biotechnology has been used for almost 30 years to improve crops through the introduction of new genes (i.e., transgenes), and more recently crops are being improved using gene editing (e.g., CRISPR/Cas9, etc.) (Chen et al., 2019). Unlike conventionally bred crops, plants developed using genetic modification have been required to undergo risk assessments prior to environmental release or commercialization for food and feed use (Brune et al., 2021).

Risk assessment is used to evaluate the impact of biotech crops on the environment, as well as human and animal safety when these products are used for food or feed (Waters et al., 2021). Risk assessments utilize established problem formulation criteria to identify and evaluate the likelihood of potential risks based on hypotheses of hazard and exposure (Devos et al., 2019). Rather than using the risk assessment process as a catch-all to characterize every and all possible adverse effect, risk assessment can apply experience and knowledge gained from breeding and 30 years of development, regulatory review, and use of biotech crops to become more efficient without undermining risk management decisions (Anderson et al., 2021; Brune et al., 2021). Improvements to the risk review process will benefit the entire agricultural industry by lowering costs associated with product development and promoting technology adoption by farmers, food processors, and even consumers, without impacting on the safety of new crops. Our experience has identified genetic stability as an area where risk analysis could be more efficient, primarily when applied to vegetatively propagated plants.

As part of the risk assessment for biotech crops, regulatory agencies require an extensive characterization of the product, including molecular details of inserted DNA and its location in the genome (EFSA, 2011). In addition, regulatory agencies also require an evaluation of genetic stability to ensure that introduced traits are stably inherited in progeny plants. The regulatory requirement to include genetic stability data in the risk assessment review for biotech crops comes from the CODEX Alimentarius guideline for foods derived from modern biotechnology, which states that molecular characterization of inserts in genetically modified plants should demonstrate that “…all expressed traits are expressed and inherited in a manner that is stable through several generations” (FAO/WHO, 2009). As most national food safety agencies align with CODEX, the requirement for genetic stability data is widely adopted for biosafety reviews of new biotech food crops.

Genetic stability as stated in CODEX includes 1) expression of the trait, and 2) heritability of the trait. In this review, we focus primarily on heritability of the trait as an indication of stable transformation of introduced DNA. Whether defined as expression or heritability, genetic stability does not inform on environmental impact or food/feed safety of a product (Anderson et al., 2021; Brune et al., 2021). Evaluation of genetic stability is a quality control measure for developers to ensure that commercialized products have the traits they claim.

Here we document over 20 years’ experience working with transgenic potato varieties to further substantiate that vegetative propagation of potato plants is genetically stable. Applying the experience and knowledge gained from this work would improve the efficiency of the regulatory review process. We recommend that automatic requirements for genetic stability data be removed from the risk assessment for vegetatively propagated biotech crops.

## 2 Vegetatively propagated crops are genetically stable

Conventional breeding practices that rely on sexual reproduction contribute to the maintenance of genetic heterozygosity within crop populations where meiosis and gamete fertilization have the potential to alter the chromosomal makeup of the cell during segregation and recombination. However, even for row crops developed using biotechnology and propagated by seed, newly inserted genes have been shown to be inherited in a stable and consistent manner similar to endogenous genes, across multiple generations (Privalle et al., 2020).

Crops such as banana, citrus, cassava, potato, and strawberry are vegetatively propagated for commercial production in order to fix desirable genotypes within cultivated varieties. Vegetative propagation circumvents challenges in the breeding process, such as self-incompatibility and inbreeding depression that have the potential to cause the loss of desirable traits (McKey et al., 2010). Vegetative propagation is considered an advantage for food production where desirable characteristics are maintained by avoiding meiosis, segregation, and homologous recombination that would introduce genetic variation in progeny plants. Vegetative propagation thus conserves the quality of planting material through multiple years of propagation (McKey et al., 2010). Examples include, the Russet Burbank potato variety, which is widely grown in the United States and has been continuously propagated for over one hundred years while maintaining genetic integrity and trait quality (Brown, 2015); and citrus trees, which have been vegetatively propagated, to maintain desirable traits, as clones or apomictic seed for several hundred years (Wu et al., 2018). For vegetatively propagated crops, detectable polymorphisms or epigenetic changes resulting in unwanted traits are eliminated from commercial production fields in order to maintain integrity of desirable genotypes (McKey et al., 2010).

**FIGURE 1:**
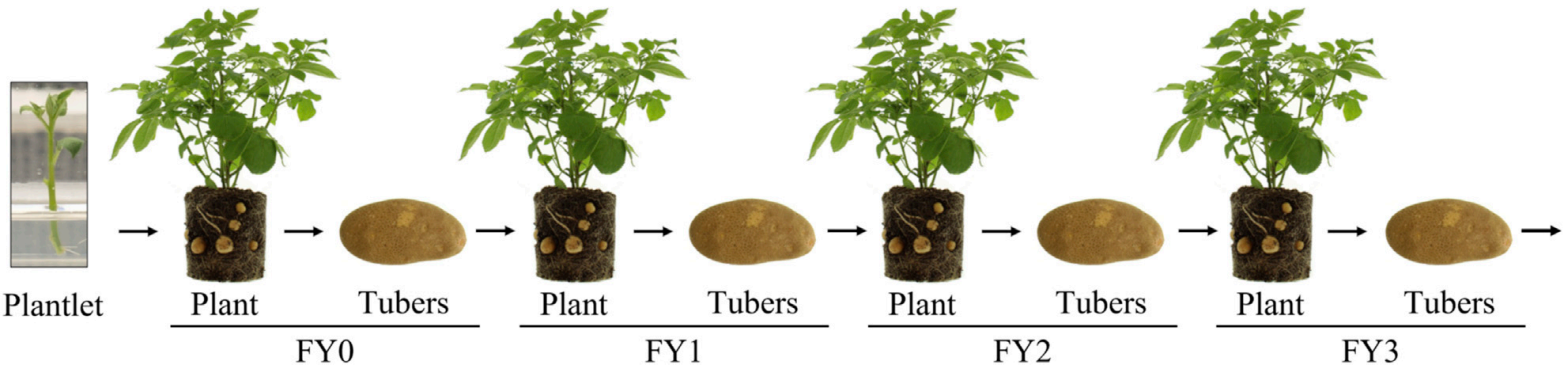
Vegetative propagation of potatoes. Tissue culture plantlets are transferred to soil or grown in hydroponic systems to produce mini-tubers, designated FY0. FY0 tubers are planted to produce FY1 plants and tubers. FY1 tubers are planted to produce FY2 plants and tubers, and so on. FY1, FY2, and FY3 tubers are typically replanted for tuber seed production. FY4, FY5 and FY6 tubers are typically sold commercially. Genetic stability data from 16 biotech potato events were collected on tubers from the vegetative propagation designated FY2.

Applying genetic stability data requirements [i.e., “inherited in a manner that is stable through several generations” (FAO/WHO, 2009)] to the safety assessment of vegetatively propagated crops raises questions and presents challenges for data collection and interpretation. For example, how does one define “inheritance” or “generation” in vegetative propagation? Any attempt to delineate a generation in a vegetatively propagated crop leads to an arbitrary classification. For example, tubers, which are the vegetative propagule of potatoes, are given a field year designation such as field year 1 (FY1). Designations are not universal and vary by geography, but are used to track vegetative propagations and are different from seed crop generations, which are the result of crosses between parent plants or self-pollination. Prior to field release, disease-free potato plantlets from tissue culture are used to produce small tubers (mini-tubers) that are designated as FY0 (Figure 1). The FY0 tubers are planted in the field and the resulting plants and tubers are designated FY1. FY1 to FY3 tubers are used primarily for commercial tuber propagation, while FY4 to FY6 tubers are sold to potato farmers for commercial crop production (Bohl and Johnson, 2010). The potato propagation pipeline is constantly replenished from tissue-culture, disease-free mother plants (Bohl and Johnson, 2010).

Published results showing stability in transgenic, vegetatively propagated crops include only a limited number of examples [i.e., sugarcane (Caffall et al., 2017; Yao et al., 2017), apple (Borejsza-Wysocka et al., 2010), pear (Lebedev, 2019), and apomictic rice (Liu et al., 2023)]. The scarcity of published results showing genetic stability in vegetatively propagated crops is presumably because researchers consider these crops genetically stable.

**FIGURE 2:**
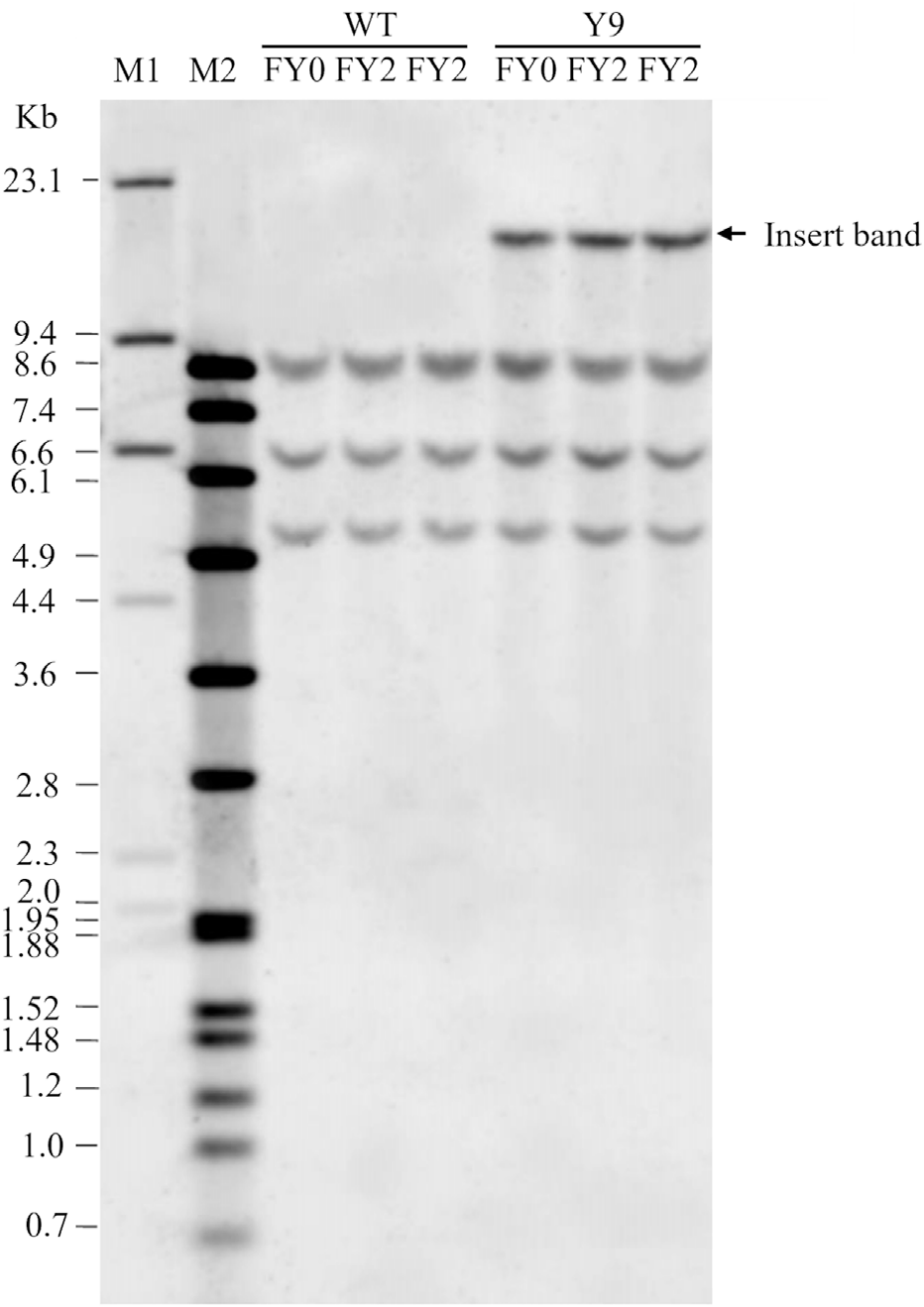
Genetic stability data. Figure adapted from (16). Southern blot result showing the stability of the inserted DNA in the Y9 potato event following three vegetative propagations. FY0 is propagation 1, FY1 (not shown) is propagation 2, and FY2 is propagation 3. The insert band is visible in all Y9 event samples demonstrating the genetic stability of vegetative propagation. The WT sample is a negative control and does not have the insert band. M1 and M2 are DNA molecular weight markers. Kb is size in kilobases.

To address the requirements of various global regulatory agencies, data were collected to demonstrate the genetic stability of transgenes from sixteen potato (*Solanum tuberosum*) varieties (Clark and Collinge, 2013; Clark et al., 2014; Spence et al., 2015; Pence et al., 2016) (example shown in Figure 2). In total, the data have been reviewed by eighty, independent scientific reviewers (Table 1). These reviewers assessed the data for environmental, food, and feed safety, and all concluded that transgenes in vegetatively propagated potatoes are stable and not a safety concern. Based on these results of genetic stability in vegetatively propagated potatoes, some regulatory agencies have begun to reconsider making genetic stability data an automatic requirement for vegetatively propagated crops (Burzaco, 2019).

**TABLE 1:** Genetic stability data from 16 potato events submitted for regulatory approvals.

Event	Data type	Regulatory agency^a^	Reference
E12	Southern blot	USDA, FDA, FSANZ, HC, CFIA, MHLW, MAFF, DOB, COFEPRIS, BPI, SFA	Clark and Collinge (2013)
E24	Southern blot	USDA, FDA	Clark and Collinge (2013)
F10	Southern blot	USDA, FDA, FSANZ, HC, CFIA, COFEPRIS	Clark and Collinge (2013)
F37	Southern blot	USDA, FDA	Clark and Collinge (2013)
J3	Southern blot PCR	USDA, FDA, FSANZ, HC, CFIA, COFEPRIS	Clark and Collinge (2013)
J55	Southern blot PCR	USDA, FDA, HC, CFIA	Clark and Collinge (2013)
J78	Southern blot PCR	USDA, FDA	Clark and Collinge (2013)
G11	Southern blot	USDA, FDA	Clark and Collinge (2013)
H37	Southern blot	USDA, FDA	Clark and Collinge (2013)
H50	Southern blot	USDA, FDA	Clark and Collinge (2013)
V11	Southern blot	USDA, FDA, FSANZ, HC, CFIA	Spence et al. (2015)
W3	Southern blot	USDA	Clark et al. (2014)
W8	Southern blot	USDA, FDA, EPA, FSANZ, HC, CFIA	Clark et al. (2014)
X17	Southern blot	USDA, FDA, EPA, FSANZ, HC, CFIA, MHLW, MAFF, DOB, COFEPRIS, BPI, SFA	Pence et al. (2016)
Y9	Southern blot	USDA, FDA, EPA, FSANZ, HC, CFIA, MHLW, MAFF, DOB, COFEPRIS, BPI, SFA	Pence et al. (2016)
Z6	Southern blot	EPA, FSANZ, HC, MHLW, MAFF	Z6 Genetic Stability Report (J. R. Simplot Company; unpublished)

^a^ USDA, United States Department of Agriculture; FDA, United States Food and Drug Administration; EPA, United States Environmental Protection Agency; FSANZ, Food Standards Australia/New Zealand; HC, Health Canada; CFIA, Canadian Food Inspection Agency; MHLW, Ministry of Health, Labour and Welfare (Japan); MAFF, Ministry of Agriculture, Forestry and Fisheries (Japan); DOB, Department of Biosafety (Malaysia); COFEPRIS, Federal Commission for Protection Against Sanitary Risks (Mexico); BPI, Bureau of Plant Industry (Philippines); SFA, Singapore Food Agency.

## 3 Improvements to the risk assessment of vegetatively propagated crops

Data on genetic stability do not necessarily inform on the safety of the transformed event. In almost all crops, whether conventionally bred or developed using biotechnology, if a new trait is not genetically stable the variety would not be commercialized.

Plants developed using biotechnology are not necessarily less stable than plants developed through conventional breeding, or even than wild relatives (Privalle et al., 2020). While molecular characterization of the inserted DNA is important for food safety assessments, genetic stability data should only be required when there is an identified pathway to harm. One possible pathway to harm that requires knowledge of genetic stability data is a loss-of-function trait that if unstable may reintroduce a health risk—for example, if genetic instability were observed in the silenced expression of solanidine glucosyltransferase in the high glycoalkaloid-containing “Lenape” potato variety (McCue et al., 2003). Genetic stability data for traits that pose this type of risk could be requested by regulators as a condition of approval for purposes of risk management.

The International Union for the Protection of New Varieties of Plants (UPOV) maintains a system for plant variety protection with certain data requirements to show that new plant varieties are distinct, uniform, and stable (DUS) (UPOV, 2002). However, UPOV does not require stability data for potato variety registration when progeny plants are uniform. The UPOV DUS guidelines state that the level of variation within self-pollinated and vegetatively propagated varieties is relatively low (UPOV, 2002), and that when a variety has been shown to be uniform, it can also be considered stable (UPOV, 2011). Building on this understanding of uniformity and stability, the UPOV *S*. *tuberosum* Testing Guideline (TG/23/6) notes that when a potato variety has been shown to be uniform, it can be considered stable and no tests for stability need to be performed (UPOV, 2004). Uniformity is an integral part of the line selection process for new potato varieties.

The experience and knowledge gained working for the past 20 years with transgenic potatoes has demonstrated the stable presence of inserted DNA and consistent performance of introduced traits following vegetative propagation, as expected. The conclusion that vegetative propagation is genetically stable applies to all vegetative crops whether developed by breeding, biotechnology, or gene editing. Requiring data that does not address safety questions for regulatory approval of vegetatively propagated crops adds unnecessary burden to an already long list of data required by regulatory agencies for risk assessment review. By removing requirements for unnecessary data, such as genetic stability data for vegetatively propagated crops, the efficiency of obtaining biotech approvals can be improved and regulatory costs reduced.

## 4 Discussion and actionable recommendations

Updates to regulatory guidance and policies is needed as new crops are improved, new technologies developed, and experience in assessing biotech crops grows. If guidance is not kept current, data requirements can result in increased regulatory burden for both developers and regulatory agencies. As an example, the rapid adoption of new gene editing technologies has left many agencies struggling to update their regulatory policies and guidance to keep pace with the development of new traits. This results in regulatory backlogs, which delay the launch of new products and prevent access to beneficial technologies for farmers, processors, consumers, and the environment.

Regulatory requirements for genetic stability data for vegetatively propagated biotech crops are not supported by science and should not be a requirement for risk assessment unless a plausible pathway to harm is identified. After 30 years of experience evaluating biotech crops, it is appropriate for regulatory authorities to eliminate data requirements that do not address environmental, food, or feed safety concerns.

By applying knowledge gained from the review of biotech products over the past three decades, regulatory agencies can reduce the regulatory burden of future biotech products without reducing the robustness of the safety review. Agencies can make an informed decision to remove this requirement based on known genetic stability of vegetatively propagated crops. As an initial step, regulators should consider removing requirements for genetic stability for vegetatively propagated biotechnology crops as part of the safety review. Regulatory authorities are encouraged to work with CODEX Alimentarius to clarify that stability assessments recommended in CAC/GL 45-2008 are not necessary for vegetatively propagated plants.
